# Supplementary material for: Conformational Plasticity-Rigidity Axis of the Coagulation Factor VII Zymogen Elucidated by Atomistic Simulations of the N-Terminally Truncated Factor VIIa Protease Domain
Source: Biomolecules. 2021 Apr 8;11(4):549. doi: 10.3390/biom11040549 (PMC8068379; doi:10.3390/biom11040549)
Supplement: Supplementary file 1 [file biomolecules-11-00549-s001.pdf]

SUPPLEMENTARY MATERIALS

Conformational Plasticity-Rigidity Axis of the Coagulation Factor VII Zymogen Elucidated by Atomistic Simulations of the N-Terminally Truncated Factor VIIa Protease Domain

Jesper J. Madsen [1,*] and Ole H. Olsen [2,*]

[1] Global and Planetary Health, College of Public Health, University of South Florida, Tampa, FL 33612, USA

[2] Novo Nordisk Foundation Center for Basic Metabolic Research, Section for Metabolic Receptology, University of Copenhagen, Blegdamsvej 3b, DK-2200 Copenhagen N, Denmark

* Correspondence: jespermadsen@usf.edu (J.J.M.); oho@sund.ku.dk (O.H.O.)

$C_\alpha - \mathrm{RMSD}$ (Å)											
$\langle \mathrm{FVIIa} \rangle_{MD}$	0										
$\langle \mathrm{FVIIa\text{-}desIVG} \rangle_{MD}$	0.936	0									
$\langle \mathrm{FVIIa}_{VYT} \rangle_{MD}$	1.860	1.955	0								
$\langle \mathrm{FVIIa}_{VYT}\text{-desIVG} \rangle_{MD}$	1.962	1.891	0.544	0							
$\langle \mathrm{Trypsin} \rangle_{MD}$	2.405	2.530	2.328	2.435	0						
$\langle \mathrm{Trypsin\text{-}desIVG} \rangle_{MD}$	2.495	2.491	2.368	2.368	0.775	0					
$\langle \mathrm{Trypsinogen} \rangle_{MD}$	3.111	3.003	2.578	2.410	1.276	0.637	0				
FVIIa^{Xtal}	2.198	2.320	1.483	1.628	2.860	2.953	3.104	0			
$\mathrm{FVIIa}_{VYT}{}^{Xtal}$	1.937	2.118	0.802	1.063	2.447	2.548	2.762	1.341	0		
$\mathrm{Trypsin}^{Xtal}$	2.980	3.128	2.371	2.492	0.735	1.186	1.464	2.798	2.404	0	
$\mathrm{Trypsinogen}^{Xtal}$	3.225	3.146	2.678	2.519	1.437	1.210	1.288	3.072	2.738	1.290	0

Table S1: RMSD values from MD trajectories are reported with reference to the averaged structure over the whole trajectory. RMSD is calculated by the align command in PyMOL without outlier rejection.

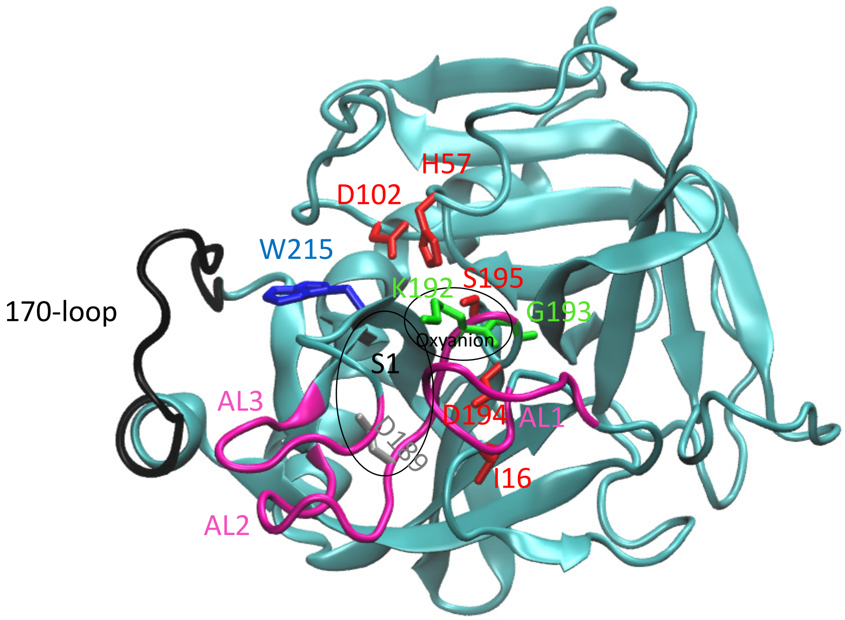

Figure S1: FVIIa (PDB ID: 1dan_H) shown as ribbon diagram with certain key loops (170-loop, AL1-3) and amino acids marked with colored labels. The active site triad consists of D102, H57 and S195 (red). The oxyanion hole consists of K192 and G193 (green). The E/Z-E*/Z* conformation is determined by W215 through its distance to H57. The S1 pocket is indicated by the gray circle with D189 placed at the bottom of the pocket.

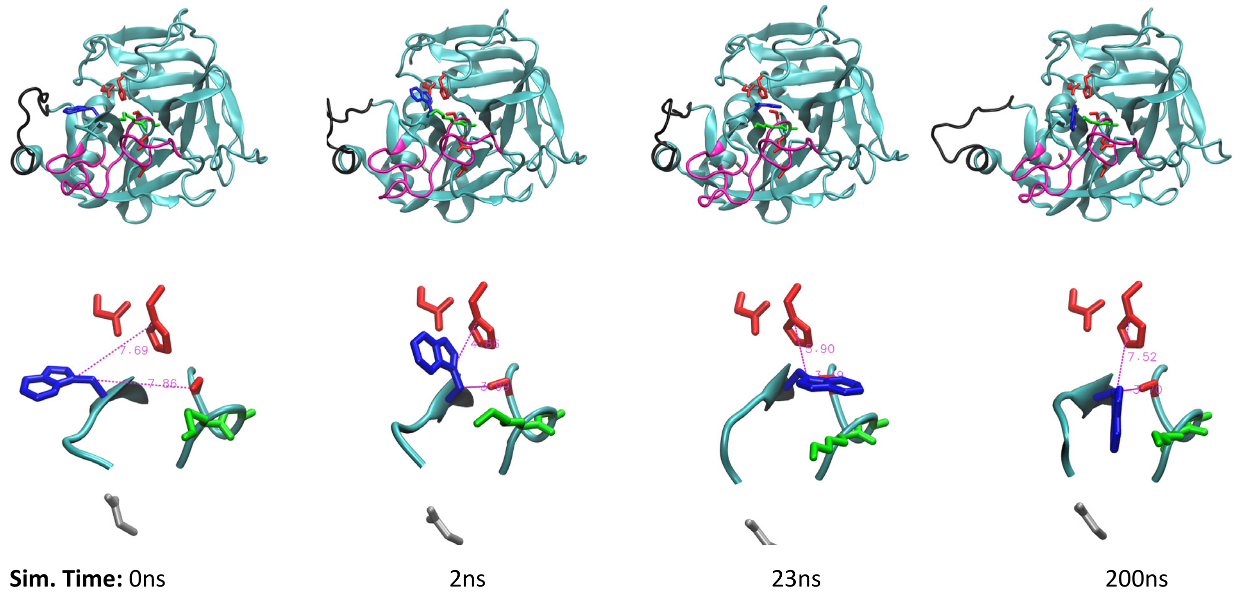

Figure S2: Structure of FVIIa (top panels) and a closeup view of the region determining the E/Z-E*/Z* conformation (bottom panels) at different stages during the simulation. The graphical details including coloring are consistent with the definition shown in Figure S2.

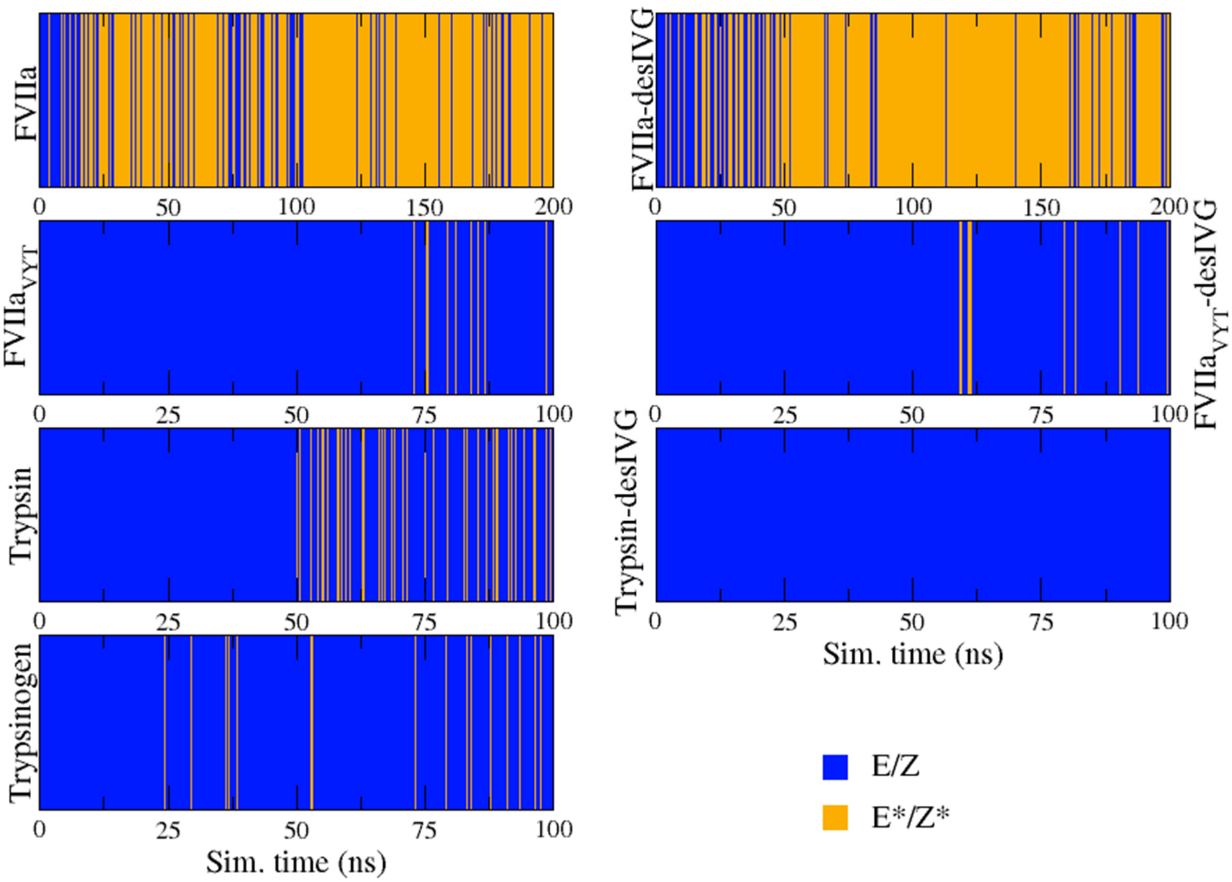

Figure S3: Time evolution of E/Z-E*/Z* conformational transitions during the simulations.

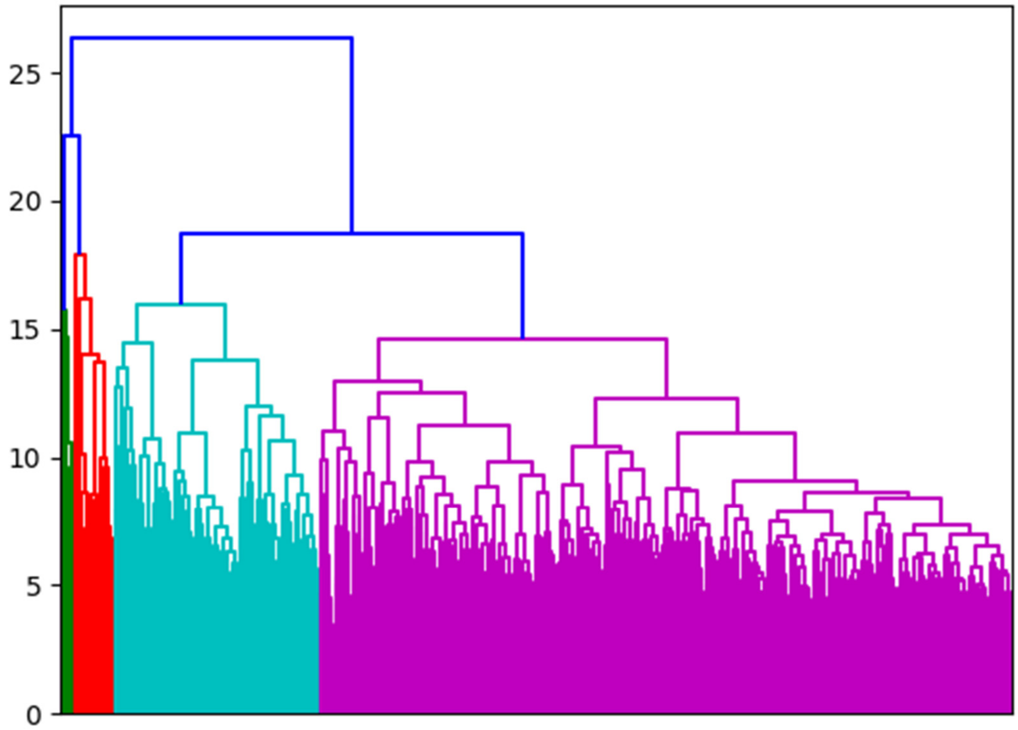

Figure S4: Dendrogram of RMSD-based hierarchical clusters from the simulated ensemble of FVIIa. Agglomeration is performed using the average/UPGMA linkage approach.

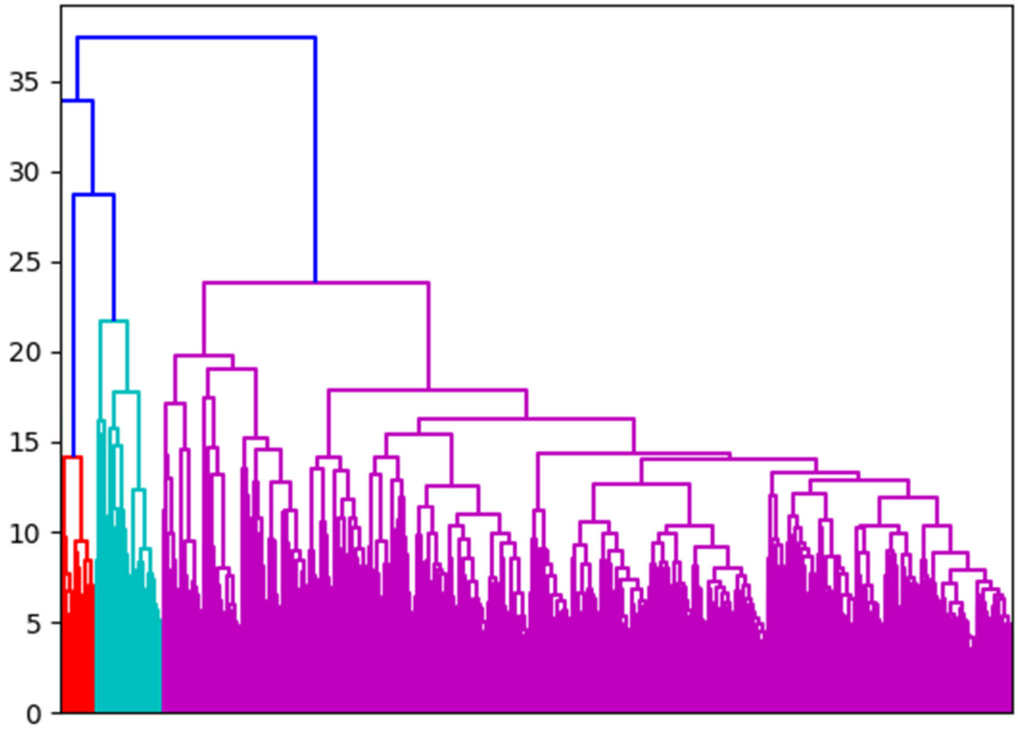

Figure S5: Dendrogram of RMSD-based hierarchical clusters from the simulated ensemble of FVIIa-desIVG. Agglomeration is performed using the average/UPGMA linkage approach.

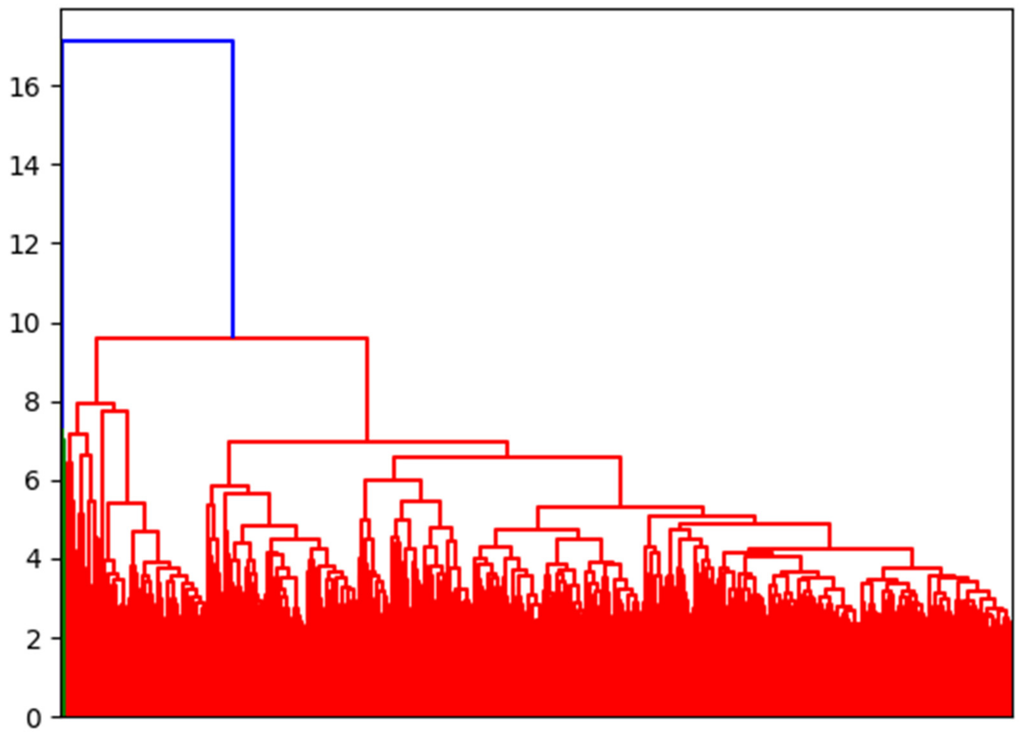

Figure S6: Dendrogram of RMSD-based hierarchical clusters from the simulated ensemble of FVIIa$_{VYT}$. Agglomeration is performed using the average/UPGMA linkage approach.

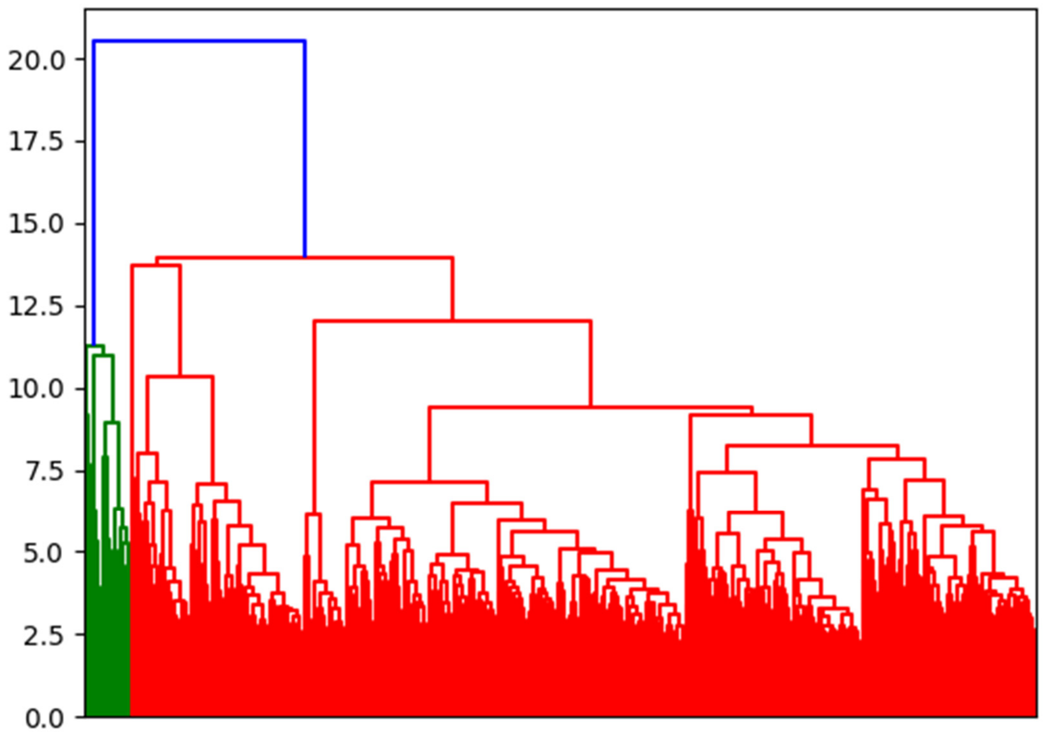

Figure S7: Dendrogram of RMSD-based hierarchical clusters from the simulated ensemble of FVIIa$_{VYT}$-desIVG. Agglomeration is performed using the average/UPGMA linkage approach.

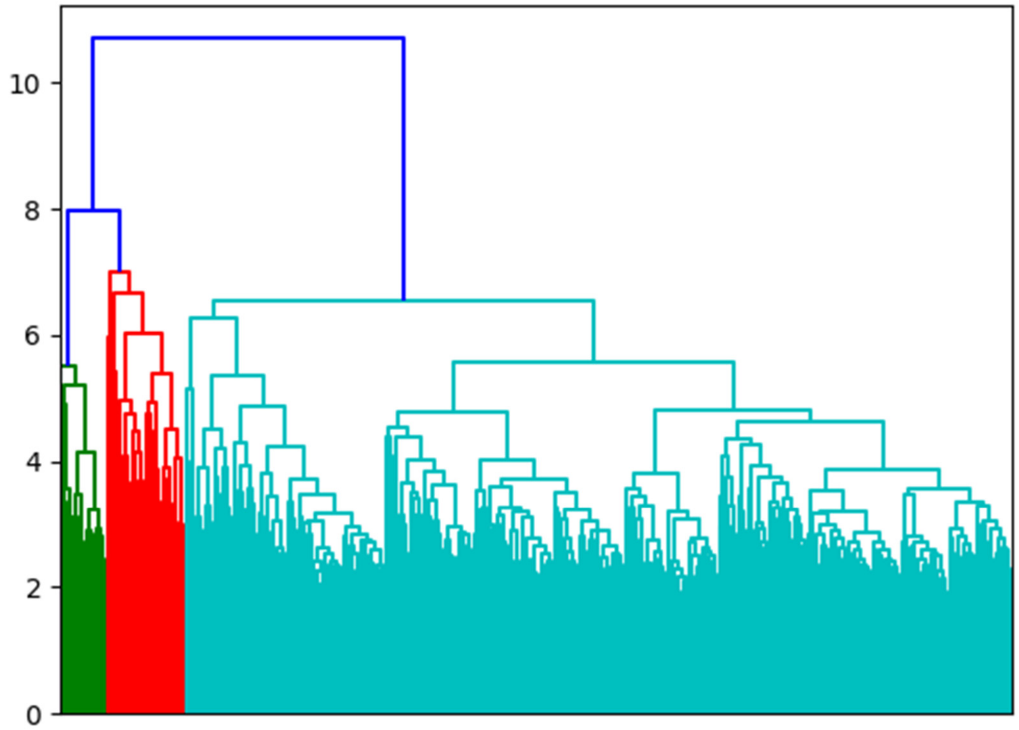

Figure S8: Dendrogram of RMSD-based hierarchical clusters from the simulated ensemble of trypsin. Agglomeration is performed using the average/UPGMA linkage approach.

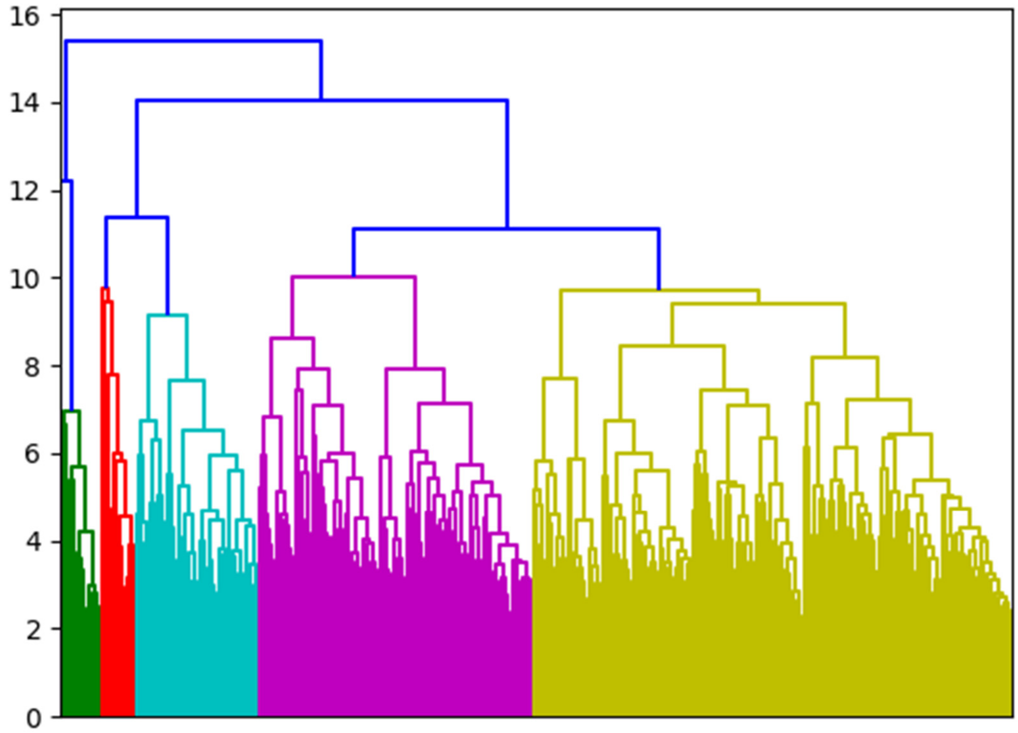

Figure S9: Dendrogram of RMSD-based hierarchical clusters from the simulated ensemble of trypsin-desIVG. Agglomeration is performed using the average/UPGMA linkage approach.

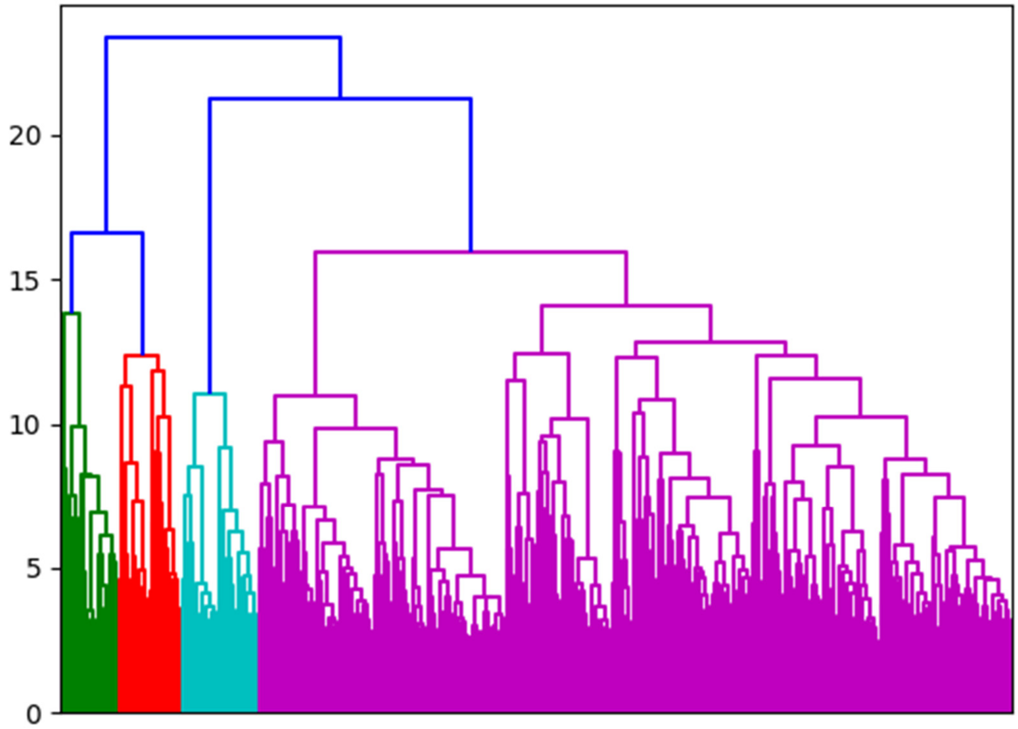

Figure S10: Dendrogram of RMSD-based hierarchical clusters from the simulated ensemble of trypsinogen. Agglomeration is performed using the average/UPGMA linkage approach.

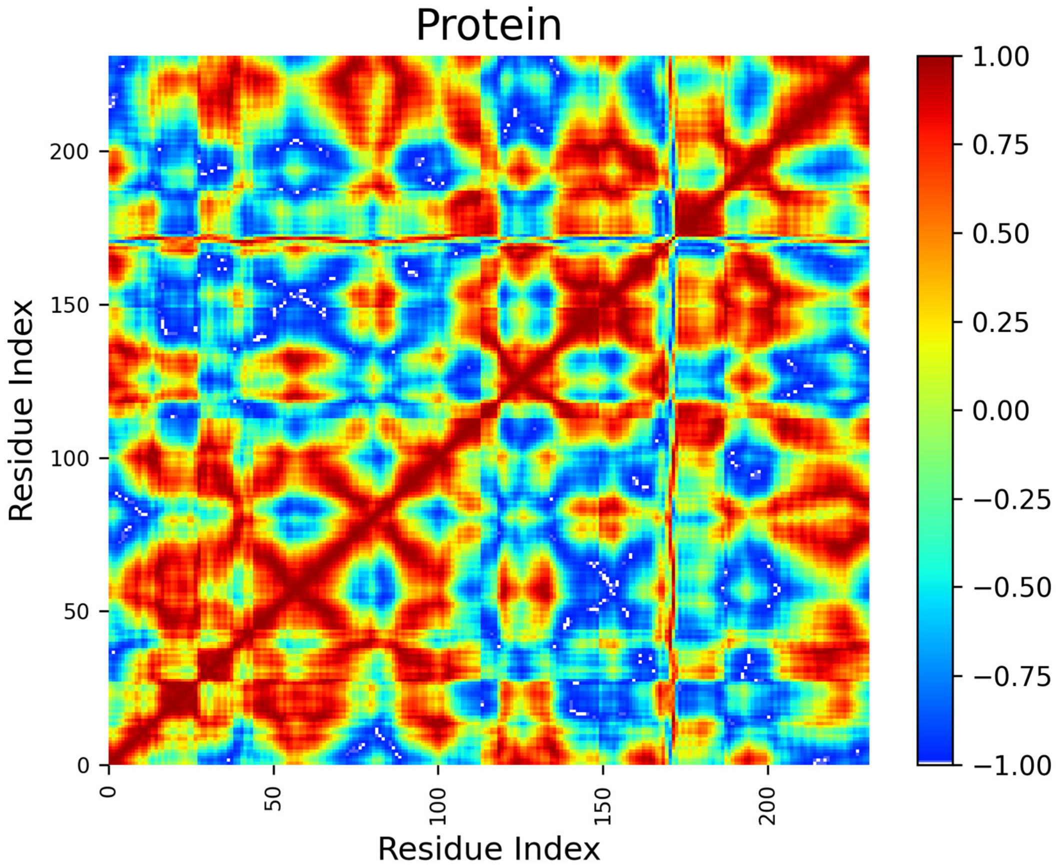

Figure S11: Heatmap of per-residue dynamic cross-correlations over the simulated trajectory of FVIIa.

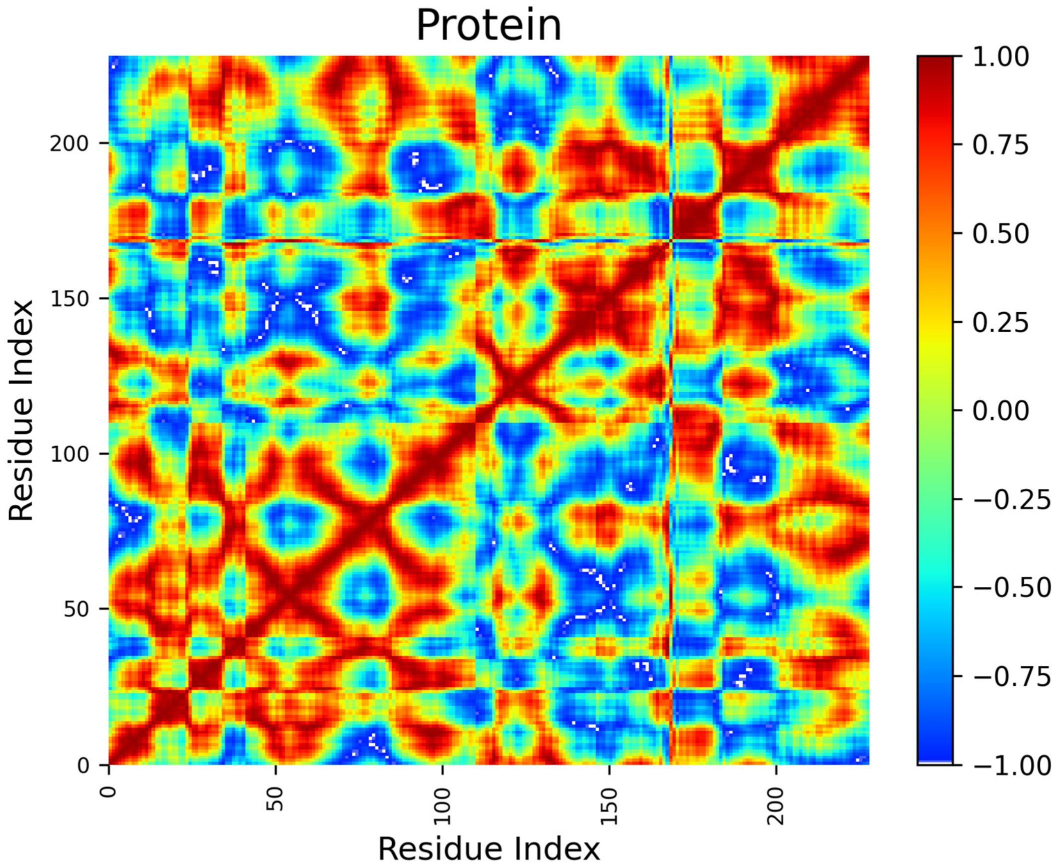

Figure S12: Heatmap of per-residue dynamic cross-correlations over the simulated trajectory of FVIIa-desIVG.

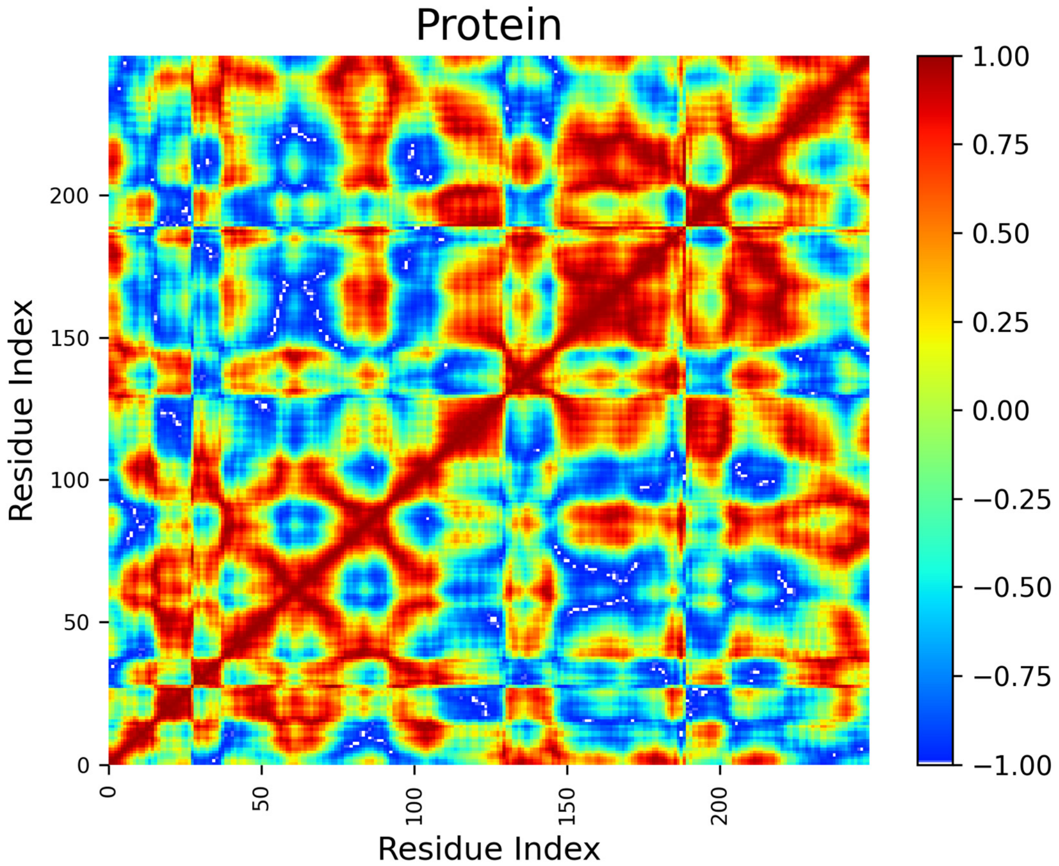

Figure S13: Heatmap of per-residue dynamic cross-correlations over the simulated trajectory of FVIIa$_{\text{VYT}}$.

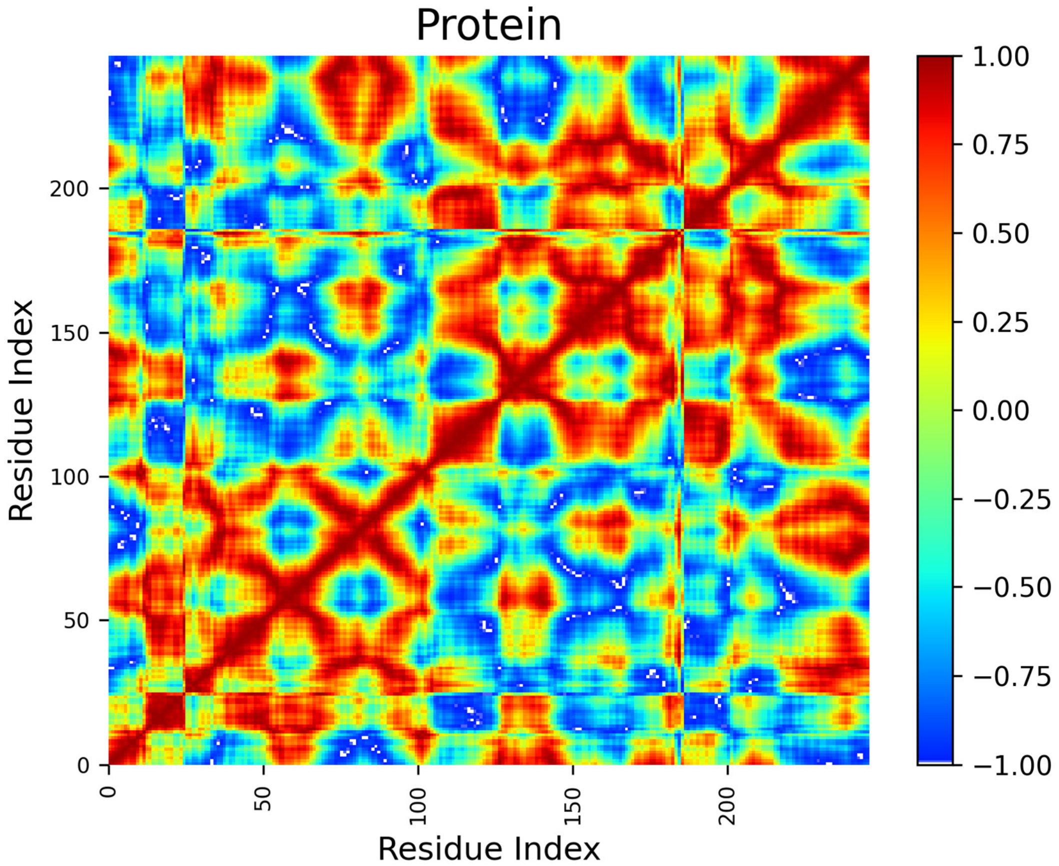

Figure S14: Heatmap of per-residue dynamic cross-correlations over the simulated trajectory of FVIIa$_{VYT}$-desIVG.

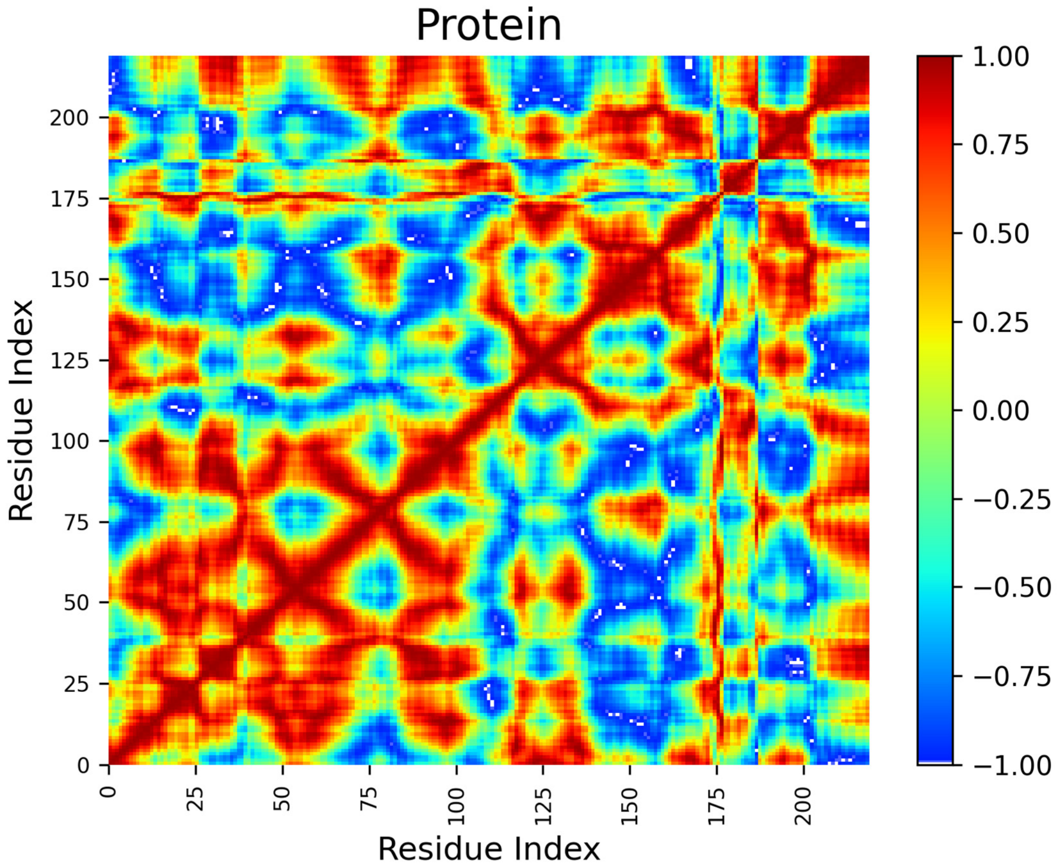

Figure S15: Heatmap of per-residue dynamic cross-correlations over the simulated trajectory of trypsin.

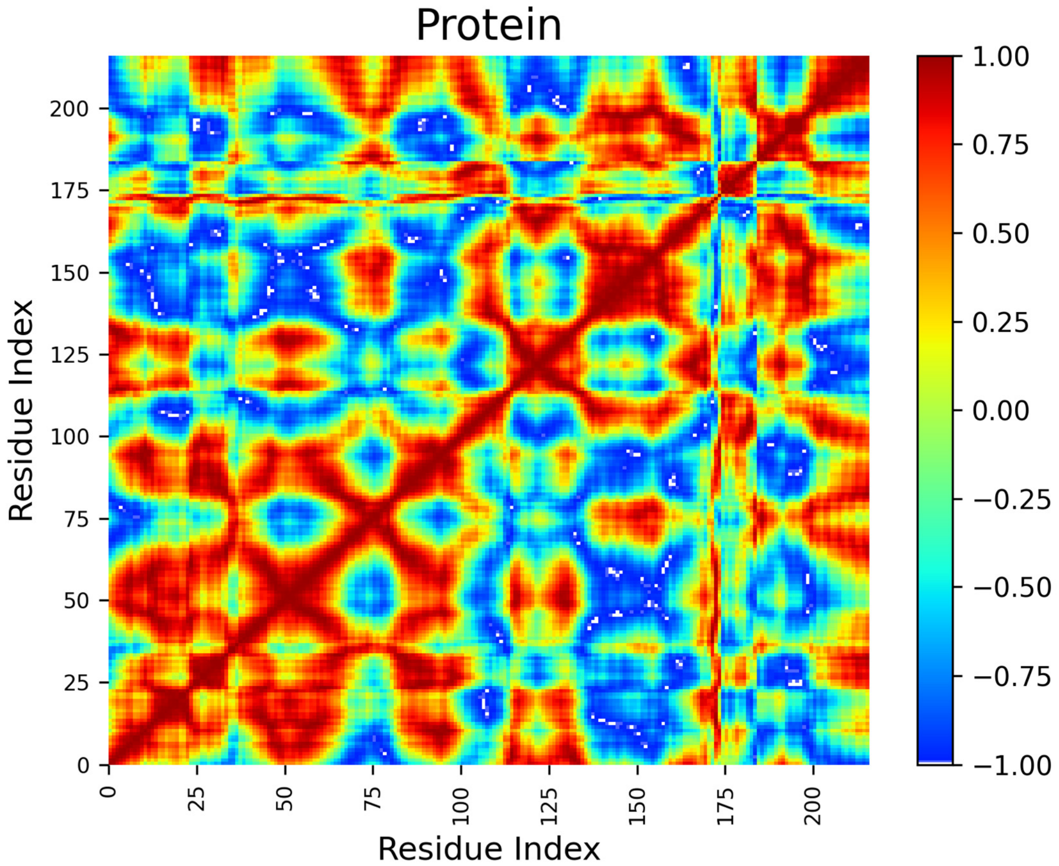

Figure S16: Heatmap of per-residue dynamic cross-correlations over the simulated trajectory of trypsin-desIVG.

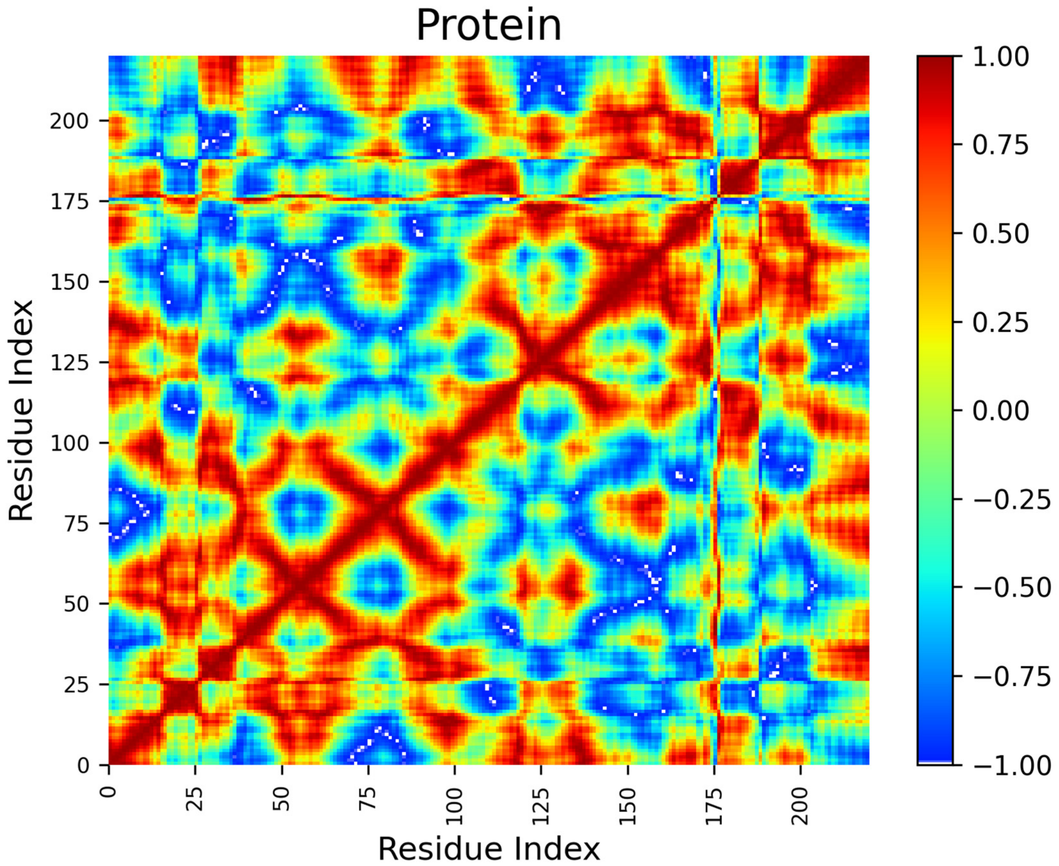

Figure S17: Heatmap of per-residue dynamic cross-correlations over the simulated trajectory of trypsinogen.
